# Serial CT changes in different components of lung cancer associated with cystic airspace in patients treated with neoadjuvant chemotherapy

**DOI:** 10.1038/s41598-021-02897-6

**Published:** 2021-12-07

**Authors:** Peipei Dou, Yankai Meng, Hengliang Zhao, Shuai Zhang, Zhongxiao Liu, Lili Zhu, Kai Xu

**Affiliations:** 1 Department of Radiology, The Affiliated Hospital of Xuzhou Medical University, 99 Huaihai West Road, Quanshan District, Xuzhou, 221000 Jiangsu People’s Republic of China; 2 Institute of Medical Imaging and Digital Medicine, Xuzhou Medical University, 209 Tongshan Road, Yunlong District, Xuzhou, 221000 Jiangsu People’s Republic of China; 3 Department of Radiology, Huaihai Hospital Affiliated with Xuzhou Medical University, Xuzhou, Jiangsu People’s Republic of China

**Keywords:** Cancer, Oncology

## Abstract

The aim of this study was to observe changes in different components (solid, cystic airspace, or entire tumor) in lung cancer associated with cystic airspace following treatment with neoadjuvant chemotherapy (NC), using computerized tomography (CT). We analyzed serial (baseline, first-time follow-up, and last-time follow-up) clinical data and CT imaging in six patients treated with NC. The diameters, areas, and volumes of different tumor components (solid, cystic airspace, and entire tumor) were measured. Delta (Δ) was used to represent changes in these parameters between two examinations: Δ1(%) represents the change from baseline to first follow-up after NC, and Δ2(%) represents the change from baseline to last follow-up after NC. We used the intra-group correlation coefficient (ICC) to test for consistency between parameters as measured by two radiologists. The diameter of solid components in all lesions showed a trend of continuous reduction compared with baseline (Δ1 ranged from − 8.3 to − 46.0%, Δ2 from − 30.8 to − 69.2%). For cystic airspace and entire tumors, different lesions showed different trends over the course of treatment. For diameter, area, and volume, Δ1 of changes in the solid component ranged from − 8.3 to − 46.9%, − 19.4 to − 70.8%, and − 19.1 to − 94.7%, respectively; Δ2 ranged from − 30.8 to − 69.2%, − 50.8 to − 92.1%, and − 32.7 to − 99.8% in diameter, area, and volume, respectively. Results were inconsistent between different components of lung cancer associated with cystic airspace that was treated with NC, but the diameter, area, and volume of solid components were continuously reduced during treatment. Furthermore, area and volume measurements showed more-significant variation than diameter measurements.

## Introduction

Lung cancer associated with cystic airspace is a unique type of lung cancer^1,2^. Tumors with mixed cystic-airspace and solid components account for about 3.7% of all lung cancers^3^. Mascalchi et al*.*^4^ classified these tumors by their different imaging manifestations into four types (I, II, III, and IV). The most common pathological categories include adenocarcinoma (AC) and squamous-cell carcinoma (SCC)^5,6^.

At present, most research on cystic-airspace lung cancer focuses on morphological features as shown on computed tomography (CT), pathological characteristics, and mechanism of tumor formation^7–10^. Some studies have reported on treatment modalities and survival outcomes^11,12^. However, few discuss the criteria by which to evaluate treatment for cystic-airspace lung cancer. Oncologists do not know whether the Response Evaluation Criteria in Solid Tumors (RECIST) apply equally to cystic-airspace lesions, or which component of the tumor (solid, cystic airspace, or entire tumor) is most suitable for evaluation. Accordingly, detailed serial changes in the solid component, cystic airspace, and total tumor might suitably indicate treatment response in this subtype of lung cancer. Furthermore, serial results of imaging parameters other than tumor diameter (i.e., tumor area or volume) at different time points (baseline, first examination, and last examination) must also be evaluated. This information will aid clinicians in evaluating treatment response and help them optimize follow-up protocol.

The aim of this study was to observe serial parameter changes in different components of lung cancer associated with cystic airspace after treatment with neoadjuvant chemotherapy (NC).

## Materials and methods

This study was conducted in accordance with the Declaration of Helsinki and approved by the Ethics Committee of Affiliated Hospital of Xuzhou Medical University (Xuzhou, China; ID No. XYFY2018-KL097-01). As it was a retrospective study, the Ethics Committee exempted it from informed-consent requirements.

### Clinical and follow-up data

Patient inclusion criteria included (1) tumor confirmed as primary lung cancer by pathological examination, (2) NC treatment, (3) no previous history of other malignant tumors or related anti-tumor treatment, and (4) consent to undergo CT imaging. Patient exclusion criteria included (1) incomplete NC treatment and (2) lack of serial CT imaging.

Forty-seven lung cancer patients with cystic airspace were treated at our institution between November 2017 and June 2020. Only 6 (12.8%) out of 47 patients who were treated with NC were analyzed. We retrieved their clinical and CT imaging data from the institution’s electronic medical record system (EMRS) and picture archiving and communication system (PACS). Their cancers were staged using the tumor–node–metastasis (TNM) system in accordance with the guidelines of the Union International Cancer Control (UICC), 8th edition^13^.

All patients were included for follow-up, which was conducted over the telephone or from the EMRS and PACS by a radiologist (P.P.D.) with 2 years’ experience in chest CT. The last follow-up date in this study was September 1, 2020. The study endpoint was changed in different components of lung cancer (solid, cystic airspace, and entire tumor) after chemotherapy.

### CT examination

We used different CT scanners in this retrospective study; detailed scanning parameters are listed in Supplementary Table 1. Scanning area was from the apex to the base of the lung at the end of inspiration. After scanning, we reconstructed the acquired images using a standard soft-tissue kernel algorithm at section thickness of 1.25 or 1.5 mm, both without overlap. All examination data were stored in Digital Imaging and Communications in Medicine (DICOM) format for analysis.

### CT imaging segmentation and data acquisition

We analyzed CT images from three examinations during NC treatment: baseline, first follow-up examination, and last follow-up examination. Maximum diameters, maximum cross-sectional areas, and volumes of different components (solid tumor, cystic airspace, and entire tumor) of the lesion were measured. The cystic airspace was defined as the gaseous-density shadow, the solid components as soft-density or ground-glass opacity (GGO), including mural nodules, cyst wall, and GGO lesions.

We imported the CT imaging data into ITK-SNAP software (version 3.8; https://www.fsf.org/) in DICOM format for image segmentation. Two radiologists (Y.K.M. and K.X.) with more than 20 years’ combined lung cancer research experience selected the region of interest (ROI) and sketched it layer by layer along the edges of the lesions. The radiologists did not know the details of clinical treatment and survival data. The average measurements they recorded were used in this study’s analysis.

**Figure 1:**
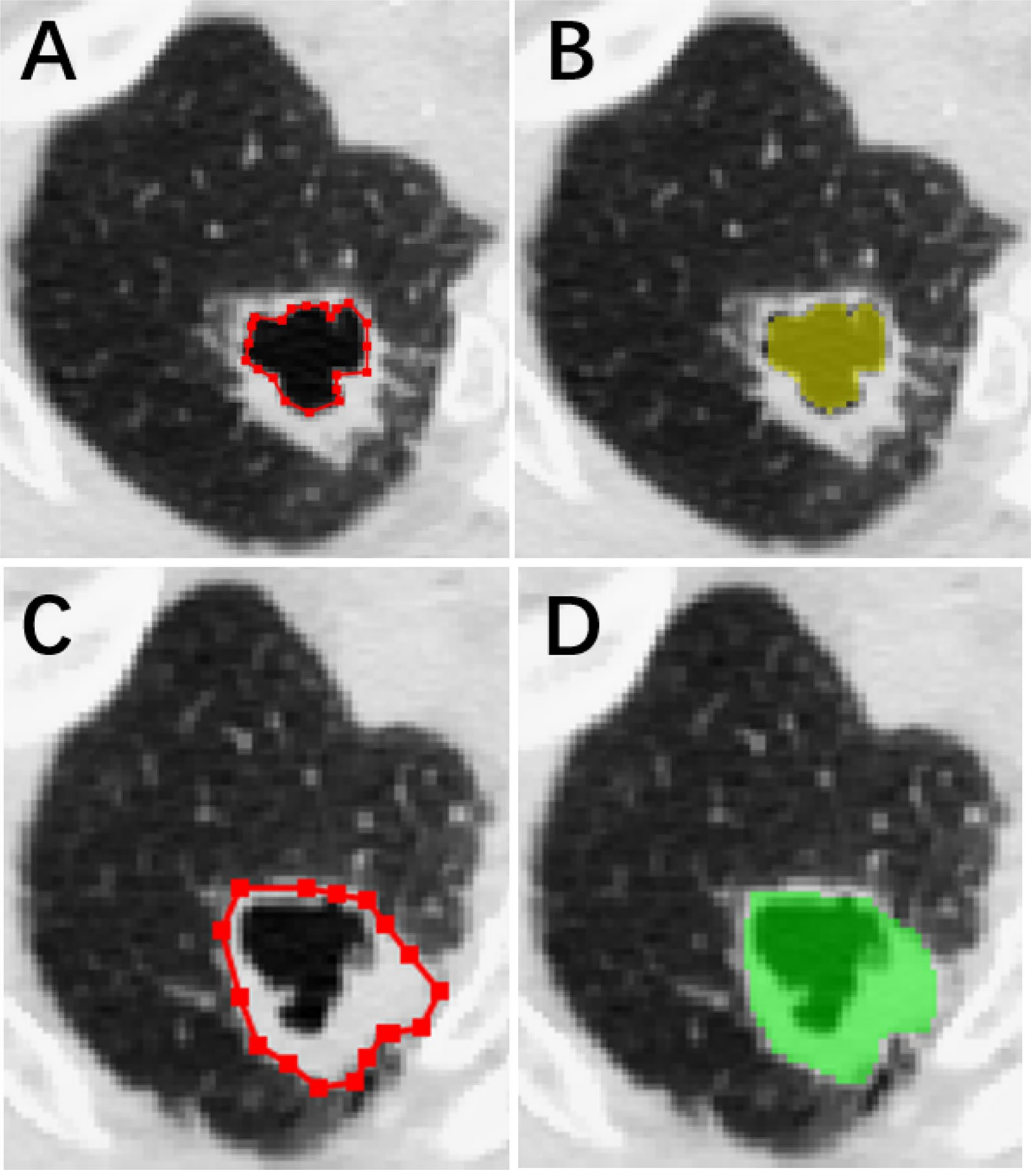
A 60-year-old man with SSC in the upper lobe of the right lung. (**A**) Edge of the cystic airspace. (**B**) Completed segmentation image (volume and area of this layer were 158.4 mm^3^ and 148.3 mm^2^, respectively). (**C**) Edge of the entire lesion. (**D**) Completed segmentation image (volume and area of this layer were 541.8 mm^3^ and 433.4 mm^2^, respectively).

Each facet of the lesion area was labeled green, while the cystic airspace was labeled yellow (Fig. 1). We computed the volumes of lesions with different components. The volume of the solid component was equal to that of the entire lesion minus the volume of the cystic airspace. To measure lesion area, the maximum cross-section of the lesion was selected and segmented along the tumor edge. After segmentation, the single-layer ROI volume was automatically output, and we obtained results after calculation according to the formula (ROI_area_ = ROI_volume_/layer thickness)^14^ (Fig. 1). The solid-component area was equal to the whole-lesion area minus the cystic-airspace area. We measured lesion diameter directly using the maximum cross-section of the lesion in accordance with the revised RECIST guideline (version 1.1)^15^.

Delta (Δ) represents changes in different parameters from examination to examination:

$$\Delta 1 \left({\%}\right)=\frac{\text{First} \; \text{Time}-\text{ Baseline}}{\text{Baseline}}; \quad \Delta 2(\%)=\frac{\text{Last} \; \text{Time}-\text{ Baseline}}{\text{Baseline}}$$

in which “Baseline” was defined as the last CT before NC, “First Time” as the initial CT examination during NC, and “Last Time” as the last CT examination after NC.

### Statistical analysis

Descriptive results were expressed as percentages (%). We used the intra-group correlation coefficient (ICC) to test the consistency of the quantitative parameters measured by the two radiologists^16^. An ICC value of 0–0.2 indicated poor consistency, 0.21–0.40 indicated average consistency, 0.41–0.60 indicated moderate consistency, 0.61–0.80 indicated relatively good consistency, and 0.81–1.00 indicated good consistency.

## Results

### Baseline clinical, laboratory, and CT findings

All six patients (all male, ages 36–73 years) had cough, and four (66.7%) had asthma. Four (66.7%) had a history of smoking. Two (33.3%) had hypertension and diabetes. Three (50%) showed elevated serum carcinoembryonic antigen (CEA) levels.

Three (50%) patients were classified as type I, one (16.7%) as type II, and two (33.3%) as type III^4^. We detected a total of six lesions, five (83.3%) of which were in the right lung. Lesion size ranged from 17.9 to 49.5 mm. Three (50%) patients had ACs, and three (50%) had SSCs. One (16.7%) patient had brain metastasis (M1 stage). Only one (16.7%) patient died during the study. Range of follow-up was 7.0–33.0 months **(**Table 1).

**Table 1:** Baseline clinical and CT findings.

Variables	Case 1	Case 2	Case 3	Case 4	Case 5	Case 6
Gender	Male	Male	Male	Male	Male	Male
Age (years)	36	59	65	60	73	64
Smoking	No	Yes	Yes	Yes	Yes	No
Hypertension	No	No	Yes	No	No	No
Diabetes	No	No	No	Yes	No	No
CEA (ng/ml)	7.8	1.7	2.9	10.7	7.5	N/A
Cough	Yes	Yes	Yes	Yes	Yes	Yes
Asthma	Yes	Yes	Yes	No	No	Yes
Chest pain	Yes	Yes	No	No	No	No
Pathological type	AC	SCC	SCC	SCC	AC	AC
Classification^a^	I	II	IV	I	IV	I
Location	Left	Right	Right	Right	Right	Right
Size (mm)^b^	34.1 × 25.2	22.4 × 15.7	49.5 × 32.1	26.8 × 23.1	39.9 × 30	17.9 × 13.4
TNM stage	T1N3M1	T1N1M0	T2N2M0	T2N1M0	T2N3M0	T1N1M0
Clinical stage	IV	II	III	II	III	II
Died	No	No	No	No	Yes	No
Follow-up time (months)	7.0	8.5	12.5	12.5	33.0	27.0

CEA: carcinoembryonic antigen (normal range, 0.00–5.00 ng/ml); AC: adenocarcinoma; SCS: squamous carcinoma; N/A: not available.

^a^Represents the Mascalchi classification of cystic lung cancer.

^b^Represents the long diameter of the lesion multiplied by the short diameter of the lesion.

### ICC of measurement parameters between the two radiologists

Supplementary Table 2 shows the ICCs of measurement parameters in lung cancer associated with cystic airspace as measured by the two radiologists. The results showed good consistency between the two physicians: ICC values of diameter, area, and volume were 0.991 (0.936–0.999), 0.980 (0.865–0.997), and 0.992 (0.946–0.999), respectively.

### Parameter changes in different components on serial CT

#### Diameters of different components

**Figure 2:**
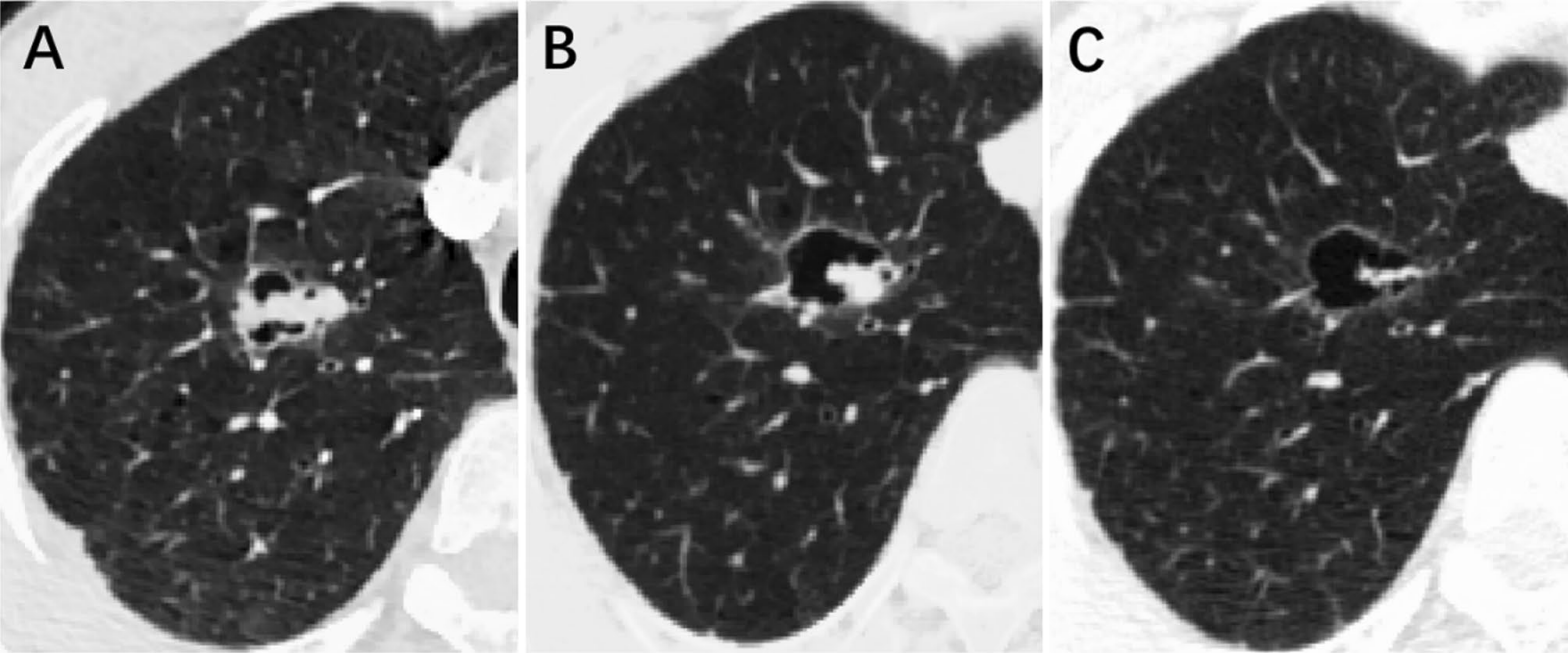
A 59-year-old male with lung cancer associated with cystic airspace (SSC). The lesion was located in the upper lobe of the right lung. (**A**) Axial-baseline CT imaging (January 21, 2020) showed solid-component and cystic-airspace components (type II). (**B**) The diameter of the solid component showed a significant decrease at the first examination during NC (April 24, 2020), while the cystic component was slightly enlarged. (**C**) The solid component continually decreased, and the lesion mainly manifested the cystic-airspace component at the last examination after NC (May 13, 2020).

The diameter of solid components in all lesions showed a trend of continuous reduction compared with baseline parameters. Δ1 ranged from − 8.3 to − 46.0% and Δ2 from − 30.8 to − 69.2% (Table 2, Fig. 2).

**Table 2:** Changes in the three different components on serial CT images.

Cases	Different components	Diameter		Area		Volume	
		Δ1 (%)	Δ2 (%)	Δ1 (%)	Δ2 (%)	Δ1 (%)	Δ2 (%)
Case 1	Solid	− 9.4	− 34.4	− 19.4	− 50.8	− 30.8	− 32.7
	Cystic airspace	− 4.6	+ 3.8	+ 39.6	+ 31.0	+ 70.0	+ 79.3
	Total lesion	− 12.6	− 38.4	− 5.3	− 45.3	− 47.1	− 49.9
Case 2	Solid	− 12.2	− 43.2	− 70.8	− 63.1	− 51.1	− 81.7
	Cystic airspace	+ 81.9	+ 115.3	+ 425.3	+ 556.5	+ 591.8	+ 812.7
	Total lesion	20.4	+ 6.9	− 20.0	+ 1.2	+ 23.6	+ 22.3
Case 3	Solid	− 17.4	− 60.9	− 37.5	− 69.3	− 36.0	− 80.8
	Cystic airspace	− 10.2	− 29.5	− 2.4	− 35.1	− 6.5	+ 52.5
	Total lesion	− 17.4	− 45.5	− 7.1	− 41.2	− 13.7	− 17.1
Case 4	Solid	− 25.0	− 69.2	− 49.4	− 92.1	− 94.7	− 99.8
	Cystic airspace	− 25.0	+ 37.1	− 38.8	+ 39.8	− 76.9	+ 15.0
	Total lesion	− 24.3	− 28.4	− 24.3	− 49.3	− 55.0	− 68.0
Case 5	Solid	− 46.9	− 62.3	− 74.3	− 86.2	− 52.6	− 90.7
	Cystic airspace	+ 27.4	− 7.5	+ 37.0	− 71.1	− 30.1	− 90.7
	Total lesion	− 38.1	− 59.6	− 62.1	− 81.7	− 73.4	− 95.2
Case 6	Solid	− 8.3	− 30.8	− 33.1	− 54.2	− 19.1	− 43.4
	Cystic airspace	+ 0.5	+ 2.5	+ 78.2	+ 104.7	+ 102.4	+ 185.4
	Total lesion	+ 21.8	+ 5.6	− 8.8	− 4.5	+ 18.0	+ 26.4

Δ1 $=\frac{\text{First} \; \text{Time}-\text{ Baseline}}{\text{Baseline}}$; Δ2 $=\frac{\text{Last} \; \text{Time}-\text{ Baseline}}{\text{Baseline}}$. Baseline: last CT before NC; First Time: initial CT examination during NC; Last Time: last CT examination in treatment. − denotes a decrease in diameter; + denotes an increase in diameter.

In terms of cystic airspace, treatment had inconsistent effects on different lesions. At the first CT examination during treatment, the cystic airspace of only three lesions had decreased (Δ1 ranged from − 4.6 to − 25.0%), whereas that of the other three lesions had increased (Δ1 ranged from 0.5 to 81.9%). At the last CT examination after treatment, the cystic airspaces of only two lesions were reduced (Δ2, − 7.5% and − 29.5%).

**Figure 3:**
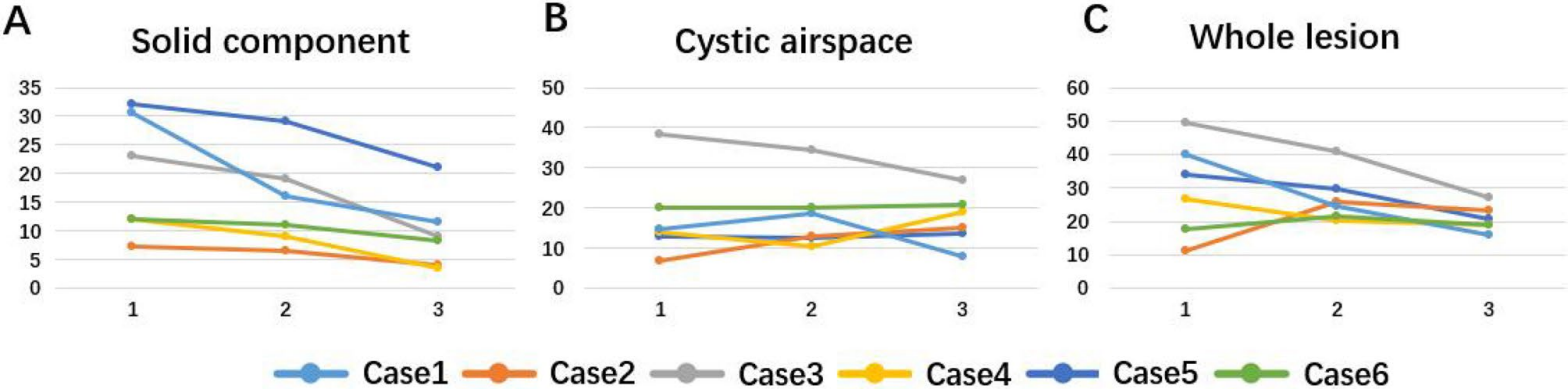
Changes in maximum diameter of different components after NC in six cases. The first abscissa represents the initial CT examination (Baseline) before NC, the second abscissa represents the first CT examination (First Time) during NC, and the last abscissa represents the last CT examination (Last Time) after NC. (**A**) Solid component; (**B**) cystic airspace; (**C**) whole lesion.

The maximum whole-lesion diameters of four lesions were reduced at the first CT examination during treatment and at the last one afterward (Δ1 ranged from − 12.6 to − 38.1% and Δ2 from − 28.4 to − 59.6%; Supplementary Table 3, Fig. 3).

#### Areas of different components

**Figure 4:**
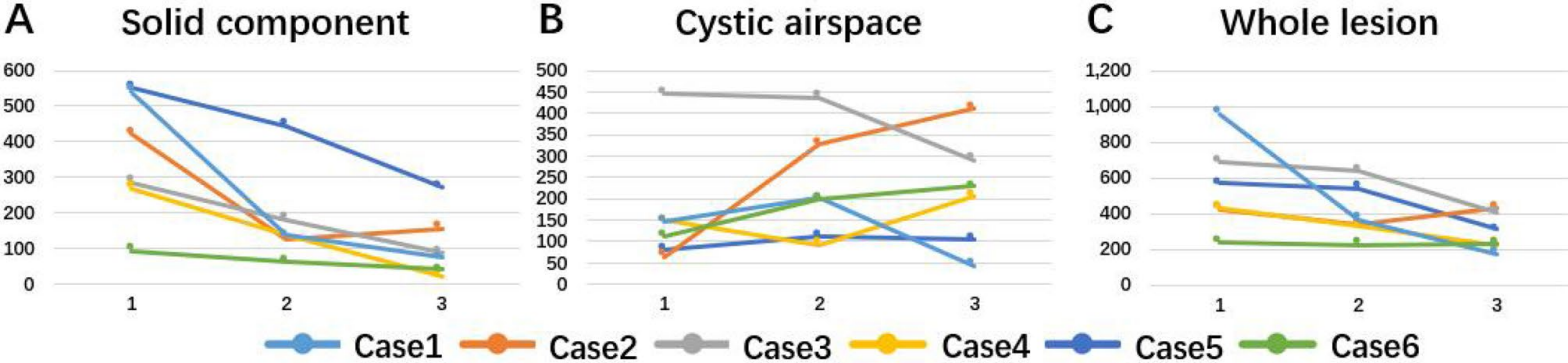
Changes in maximum area of different components after NC in six cases. The first abscissa represents the initial CT examination (Baseline) before NC, the second abscissa represents the first CT examination (First Time) during NC, and the last abscissa represents the last CT examination (Last Time) after NC. (**A**) Solid component; (**B**) cystic airspace; (**C**) whole lesion.

Compared with baseline, solid components in all six cases were reduced (Δ1 and Δ2 were − 1.94% to − 70.8% and − 50.8% to − 92.1%, respectively; Table 2). Interestingly, the cystic-airspace area of only two lesions decreased (Δ1, − 2.4% to − 38.8%; Δ2, − 35.1% to − 71.1%) while that of four increased during treatment. For Δ1, the whole-tumor area of all six lesions decreased (from − 5.3 to − 62.1%); for Δ2, the whole-tumor area of five lesions was reduced (from − 4.5 to − 81.7%; Supplementary Table 4, Fig. 4).

#### Volumes of different components

**Figure 5:**
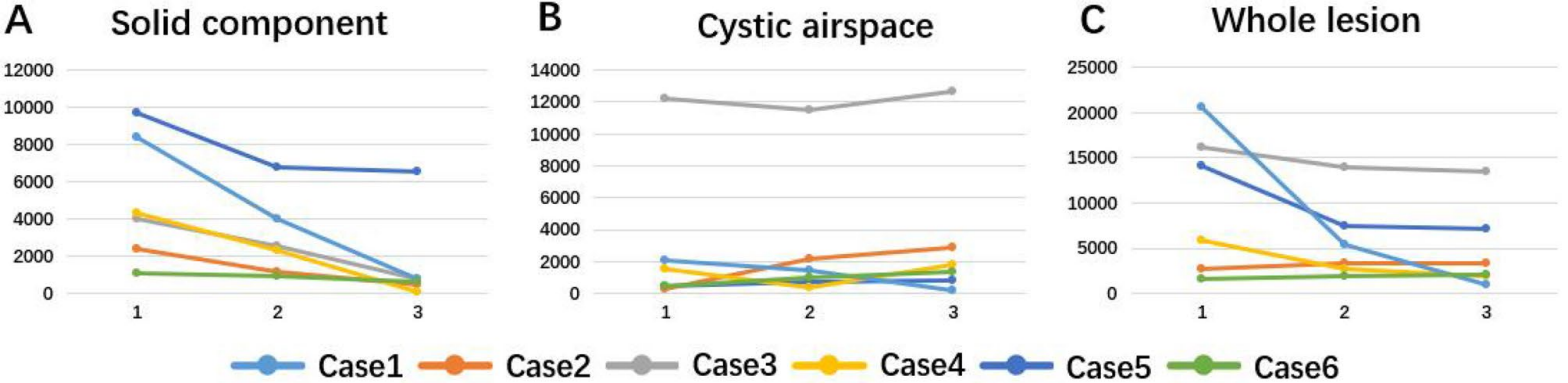
Changes in volume of different components after NC in six cases. The first abscissa represents the initial CT examination (Baseline) before NC, the second abscissa represents the first CT examination (First Time) during NC, and the last abscissa represents the last CT (Last Time) after NC. (**A**) Solid component; (**B**) cystic airspace; (**C**) whole lesion.

Similarly, solid-component volume decreased significantly in all six lesions during follow-up (Table 2). In terms of cystic-airspace volume, three lesions decreased and three increased at the first CT examination (Δ1 ranged from − 6.5 to − 76.9% and from + 70.0 to + 591.8%, respectively). However, at the last CT examination after treatment, only one lesion decreased in cystic volume (Δ2 =  − 90.7%). In terms of whole-lesion volume, four lesions decreased (Δ1 ranged from − 13.7 to − 73.4%, Δ2 from − 17.1 to − 95.2%; Supplementary Table 5, Fig. 5).

### Comparisons of different measurement parameters (diameter, area, volume) with serial CT

The magnitude of variation was larger in volume and area than in diameter (Table 2). For diameter, area, and volume, Δ1 of measured changes in the solid component ranged from − 8.3 to − 46.9, − 19.4 to − 70.8, and − 19.1 to − 94.7, respectively; while Δ2 ranged from − 30.8 to − 69.2, − 50.8 to − 92.1, and − 32.7 to − 99.8, respectively. Changes in cystic-airspace and whole-tumor parameters showed similar results.

## Discussion

In this study, we measured different components of lung cancer associated with cystic airspace. Interestingly, the trends in our results were not consistent among solid components, cystic-airspace components, and entire tumors on serial CT scan. Furthermore, area and volume showed significant changes, while diameter did not. We observed additional contrary tendencies in most lesions for solid and cystic-airspace components.

Lung cancer associated with cystic airspace is a rare manifestation of the disease. At present, CT is one of the main modalities for cancer staging and for evaluating treatment response and outcomes^17,18^. Most studies have evaluated treatment response by size of solid tumors^19,20^. Furthermore, the RECIST guideline (version 1.1) is recommended for such evaluation^15^. However, the relevant evaluation criteria for cystic lung cancer are rarely discussed, which can confuse oncologists’ attempts to assess the criteria for tumor treatment response and therefore influence treatment strategies for individual patients in clinical practice. Our results demonstrated that different measurements of tumor components showed different results and that only the solid component was reduced by NC treatment. We speculate that the change in the solid component might be more correlated with clinical treatment response. In cystic-airspace lung cancer patients treated with NC, this component was more suitable for evaluation than were the cystic-airspace component and total tumor. However, we observed only six patients, and our study did not discuss the correlations of different component results with tumor long-term outcomes. Therefore, these correlations must be further evaluated by survival outcomes in a larger cohort over a longer follow-up time. The results of such studies could help guide the treatment for lung cancer associated with cystic airspace.

Furthermore, when comparing the degrees of variation in the three measurement parameters (tumor diameter, area, and volume), we found that area and volume changes in treatment were more significant than diameter changes. Some studies show that analysis of the largest cross-sectional area and of volume might more accurately depict the burden of complex objects compared with traditional linear size measurement, which has great clinical implications^21–23^. This could be because small changes in linear size are amplified by corresponding changes in area and volume. Therefore, area and volume might more comprehensively reflect tumor burden by objective three-dimensional measurement, and so measuring these two parameters in lung cancer associated with cystic airspace could be a useful supplement to RECIST criteria. However, the measurement error of area and volume is large, and the working procedure is complicated, which might limit its clinical application.

Some studies that have investigated the formation of cystic airspace have shown that tumor cell proliferation can lead to the formation of valves in the bronchial lumen^2,24,25^. The small bronchial stenosis prevents partial air exchange, resulting in increased residual-air volume in alveolar space. In our study, some solid components decreased, while some cystic lesions increased, over the course of NC treatment. Solid and cystic-airspace component parameters had contrary tendencies in some lesions. We thought that perhaps the reduction in the solid components resulted in enlargement of the “unidirectional valves.” The parameters of the cystic-airspace component including the total tumor in the lesion were increased. Therefore, we speculate that changes in the cystic-airspace component did not accurately and objectively reflect treatment response.

This study had some limitations. First, the number of cases was small; we analyzed the serial CT images of only six patients. Second, patients’ NC regimens were inconsistent, which might have affected treatment efficacy. Furthermore, two patients were T1N1M0, and one was T2N1M0. We speculate that perhaps the oncologist used an aggressive treatment strategy in clinical practice. Third, such patients’ actual conditions and attitudes toward treatment might influence the oncologist’s treatment decisions. Finally, the pathological lung cancer types of the enrolled patients were not consistent, so we did not perform different subgroup analyses for different pathological types.

In conclusion, after neoadjuvant chemotherapy in lung cancer with cystic airspace, measurements of different components showed inconsistent results, with only the solid component reduced during treatment. Area and volume measurements changed significantly compared with diameter measurements over the course of treatment.

## Supplementary Information

Supplementary Table 1.

Supplementary Table 2.

Supplementary Table 3.

Supplementary Table 4.

Supplementary Table 5.

Supplementary Table 6.
