# Supplementary material for: Serial CT changes in different components of lung cancer associated with cystic airspace in patients treated with neoadjuvant chemotherapy
Source: Sci Rep. 2021 Dec 7;11:23544. doi: 10.1038/s41598-021-02897-6 (PMC8651644; doi:10.1038/s41598-021-02897-6)
Supplement: Supplementary file 1 — Supplementary Table 1. [file 41598_2021_2897_MOESM1_ESM.docx]

**Supplementary Table 1.** The parameters of CT scan

| CT Parameters | Value |
| --- | --- |
| The thickness of scanning (mm) | 5 |
| The interval of scanning (mm) | 5 |
| The thickness of reconstruction (mm) | 1.25/1.5 |
| The interval of reconstruction (mm) | 1.25/1.5 |
| Pulmonary window position (HU) | -600 |
| Pulmonary window width (HU) | 1200 |
| Mediastinal window position (HU) | 40 |
| Mediastinal window width (HU) | 300 |
