# Supplementary material for: Serial CT changes in different components of lung cancer associated with cystic airspace in patients treated with neoadjuvant chemotherapy
Source: Sci Rep. 2021 Dec 7;11:23544. doi: 10.1038/s41598-021-02897-6 (PMC8651644; doi:10.1038/s41598-021-02897-6)
Supplement: Supplementary file 2 — Supplementary Table 2. [file 41598_2021_2897_MOESM2_ESM.docx]

**Supplementary Table 2**. The timing of CT examination and the regimen of NC

| Cases | Baseline time | First time | Last time | Treatment regimens |
| --- | --- | --- | --- | --- |
| Case 1 | 2020/2/19 | 2020/3/21 | 2020/7/9 | Pemetrexed + cisplatin |
| Case 2 | 2020/1/21 | 2020/4/24 | 2020/5/13 | Gemcitabine + nedaplatin |
| Case 3 | 2019/9/20 | 2019/10/14 | 2020/6/16 | Paclitaxel + carboplatin |
| Case 4 | 2019/9/19 | 2019/10/21 | 2020/7/27 | Paclitaxel + nedaplatin |
| Case 5 | 2017/12/14 | 2018/2/2 | 2018/3/28 | Pemetrexed + nedaplatin |
| Case 6 | 2018/5/21 | 2018/7/20 | 2020/7/2 | Pemetrexed + cisplatin |

Note: NC means neoadjuvant chemotherapy; Baseline time was the last CT before NC; first time was the initial CT examination in NC; last time was the last CT examination after NC.
