# Supplementary material for: Serial CT changes in different components of lung cancer associated with cystic airspace in patients treated with neoadjuvant chemotherapy
Source: Sci Rep. 2021 Dec 7;11:23544. doi: 10.1038/s41598-021-02897-6 (PMC8651644; doi:10.1038/s41598-021-02897-6)
Supplement: Supplementary file 3 — Supplementary Table 3. [file 41598_2021_2897_MOESM3_ESM.docx]

**Supplementary Table 3: ICC of** [**measurement**](javascript:;) **parameters of two radiologists**

| Variables | ICC | 95% CI | |  |
| --- | --- | --- | --- | --- |
| Diameter  Area  Volume | 0.991  0.980  0.992 | | 0.936–0.999  0.865–0.997  0.946–0.999 |  |

Note: ICC, intra-class correlation coefficient; CI, confidence interval.
