# Supplementary material for: Serial CT changes in different components of lung cancer associated with cystic airspace in patients treated with neoadjuvant chemotherapy
Source: Sci Rep. 2021 Dec 7;11:23544. doi: 10.1038/s41598-021-02897-6 (PMC8651644; doi:10.1038/s41598-021-02897-6)
Supplement: Supplementary file 4 — Supplementary Table 4. [file 41598_2021_2897_MOESM4_ESM.docx]

**S**[**upplementary**](javascript:;) **Table 4**. The changes of maximum diameter with serial CT.

| Cases | Different components | Baseline (mm) | First Time  (mm) | Last Time  (mm) |
| --- | --- | --- | --- | --- |
| Case 1 | Solid | 32.0 | 29.0 | 21.0 |
|  | Cystic airspace | 13.1 | 12.5 | 13.6 |
|  | Total lesion | 34.1 | 29.8 | 21.0 |
| Case 2 | Solid | 7.4 | 6.5 | 4.2 |
|  | Cystic airspace | 7.2 | 13.1 | 15.5 |
|  | Total lesion | 21.6 | 26.0 | 23.1 |
| Case 3 | Solid | 23.0 | 19.0 | 9.0 |
|  | Cystic airspace | 38.3 | 34.4 | 27.0 |
|  | Total lesion | 49.5 | 40.9 | 27.0 |
|  | Solid | 12.0 | 9.0 | 3.7 |
| Case 4 | Cystic airspace | 14.0 | 10.5 | 19.2 |
|  | Total lesion | 26.8 | 20.3 | 19.2 |
|  | Solid | 30.5 | 16.2 | 11.5 |
| Case 5 | Cystic airspace | 14.6 | 18.6 | 13.5 |
|  | Total lesion | 39.9 | 24.7 | 16.1 |
|  | Solid | 12.0 | 11.0 | 8.3 |
| Case 6 | Cystic airspace | 20.2 | 20.3 | 20.7 |
|  | Total lesion | 17.9 | 21.8 | 18.9 |

**Note:** Baseline, the last CT before neoadjuvant chemotherapy (NC); First Time: the initial CT examination in NC. Last Time: the last CT examination in treatment. Δ1$=\frac{First Time- Baseline}{\mathrm{Baseline}}$. Δ2$=\frac{Last Time- Baseline}{\mathrm{Baseline}}$. – means diameter decreased, + means diameter increased.
