# Supplementary material for: Serial CT changes in different components of lung cancer associated with cystic airspace in patients treated with neoadjuvant chemotherapy
Source: Sci Rep. 2021 Dec 7;11:23544. doi: 10.1038/s41598-021-02897-6 (PMC8651644; doi:10.1038/s41598-021-02897-6)
Supplement: Supplementary file 5 — Supplementary Table 5. [file 41598_2021_2897_MOESM5_ESM.docx]

**S**[**upplementary**](javascript:;) **Table 5.** The change of maximum area with serial CT.

| Cases | Different components | Baseline  (mm^2^) | First Time  (mm^2^) | Last Time (mm^2^) |
| --- | --- | --- | --- | --- |
| Case1 | Solid | 550.0 | 443.2 | 270.5 |
|  | Cystic airspace | 79.3 | 110.7 | 103.9 |
|  | Total lesion | 567.2 | 537.0 | 310.2 |
| Case2 | Solid | 424.4 | 123.8 | 156.7 |
|  | Cystic airspace | 62.5 | 328.3 | 410.3 |
|  | Total lesion | 424.4 | 339.5 | 429.4 |
| Case3 | Solid | 286.0 | 178.7 | 87.7 |
|  | Cystic airspace | 447.6 | 436.7 | 290.6 |
|  | Total lesion | 689.4 | 640.3 | 405.7 |
| Case4 | Solid | 269.6 | 136.4 | 21.2 |
|  | Cystic airspace | 148.3 | 90.8 | 207.3 |
|  | Total lesion | 433.4 | 328.3 | 219.8 |
| Case5 | Solid | 539.6 | 138.5 | 74.6 |
|  | Cystic airspace | 147.4 | 202.0 | 42.6 |
|  | Total lesion | 957.6 | 362.8 | 175.3 |
| Case6 | Solid | 91.8 | 61.4 | 42.0 |
|  | Cystic airspace | 111.7 | 199.0 | 228.6 |
|  | Total lesion | 239.4 | 218.4 | 228.6 |

**Note:** Baseline, the initial CT before NC; First Time: the first CT in NC. Last Time: the last CT examination in NC. Δ1$=\frac{First Time- Baseline}{\mathrm{Baseline}}$. Δ2$=\frac{Last Time- Baseline}{\mathrm{Baseline}}$.
