# Supplementary material for: Serial CT changes in different components of lung cancer associated with cystic airspace in patients treated with neoadjuvant chemotherapy
Source: Sci Rep. 2021 Dec 7;11:23544. doi: 10.1038/s41598-021-02897-6 (PMC8651644; doi:10.1038/s41598-021-02897-6)
Supplement: Supplementary file 6 — Supplementary Table 6. [file 41598_2021_2897_MOESM6_ESM.docx]

**S**[**upplementary**](javascript:;) **Table 6.** The changes of volume with serial CT.

| Cases | Different components | Baseline (mm^3^) | First Time  (mm^3^) | Last Time  (mm^3^) |
| --- | --- | --- | --- | --- |
| Case1 | Solid | 9741.2 | 6745.4 | 6560.6 |
|  | Cystic airspace | 441.2 | 750.1 | 791.0 |
|  | Total lesion | 14182.4 | 7495.5 | 7102.2 |
| Case2 | Solid | 2417.4 | 1181.0 | 442.6 |
|  | Cystic airspace | 317.9 | 2199.1 | 2901.6 |
|  | Total lesion | 2735.3 | 3380.1 | 3344.2 |
| Case3 | Solid | 3981.8 | 2547.6 | 764.5 |
|  | Cystic airspace | 12259.2 | 11463.6 | 18700.0 |
|  | Total lesion | 16241.0 | 14011.2 | 13464.5 |
| Case4 | Solid | 43331.3 | 2293.4 | 85.1 |
|  | Cystic airspace | 1568.9 | 361.9 | 1803.7 |
|  | Total lesion | 5900.2 | 2655.3 | 1888.8 |
| Case5 | Solid | 8427.3 | 3993.9 | 787.2 |
|  | Cystic airspace | 2100.5 | 1468.2 | 196.1 |
|  | Total lesion | 20527.8 | 5462.1 | 983.3 |
| Case6 | Solid | 1112.2 | 900.2 | 629.8 |
|  | Cystic airspace | 488.3 | 988.4 | 1393.6 |
|  | Total lesion | 1600.6 | 1888.6 | 2023.5 |

**Note:** Baseline, the last CT before neoadjuvant chemotherapy (NC); First Time: the initial follow-up CT in NC. Last Time: the last follow-up CT in NC. Δ1$=\frac{First Time- Baseline}{\mathrm{Baseline}}$. Δ2$=\frac{Last Time- Baseline}{\mathrm{Baseline}}$.
